# Perceived social support and general psychological distress in the post-pandemic era: mediation of perceived stress and moderation of psychological resilience

**DOI:** 10.3389/fpsyg.2026.1939954

**Published:** 2026-10-12

**Authors:** Lixin Zhao, Zhitao Yuan, Mengyuan Yang, Xianyang Wang, Shengjun Wu

**Affiliations:** Department of Military Medical Psychology, Air Force Medical University, Xi’an, China

**Keywords:** general psychological distress, moderated mediation, perceived social support, perceived stress, psychological resilience, the post-pandemic era

## Abstract

**Background:**

In the post-pandemic era, the continuation of social distancing and the popularization of social media are changing people’s psychological states. This study examined the effect of perceived social support on general psychological distress via perceived stress, as well as the moderating role of psychological resilience.

**Methods:**

A nationwide sample of 8,131 valid responses was obtained through convenience sampling via the Questionnaire Star platform between February and March 2025. The Multidimensional Scale of Perceived Social Support (MSPSS), the Chinese Perceived Stress Scale (CPSS), the Connor–Davidson Resilience Scale (CD-RISC-10), and the 28-item General Health Questionnaire (GHQ-28) were used to evaluate the related variables. The moderated mediation model was tested using Hayes’ PROCESS macro (Version 4.3), and the bias-corrected bootstrap method was used for analysis.

**Results:**

Perceived stress was positively associated with general psychological distress (*β* = 0.3628, *p* < 0.001). The interaction between perceived social support and psychological resilience was significant in predicting perceived stress (*β* = −0.3595, *p* < 0.001) and general psychological distress (*β* = 0.0803, *p* < 0.001). Conditional indirect effects were significant at low and high levels of resilience but reversed in sign: at low resilience (*M*-1*SD*), the indirect effect was positive [0.0903, 95%CI (0.0716, 0.1105)], whereas at high resilience, it was negative.

**Conclusion:**

Perceived social support was associated with lower general psychological distress, in part through lower perceived stress; psychological resilience moderated the paths from perceived social support to perceived stress and general psychological distress. The crossover interaction at low psychological resilience suggests that perceived social support does not uniformly translate into perceived stress relief across individuals.

## 1 Introduction

The COVID-19 pandemic exerted profound impacts on the public health system. At the end of 2022, China’s National Health Commission announced the Category B, Level B management policy for COVID-19 infection, and January 2023 marked the entry into the post-pandemic era (National Health Commission of the PRC, 2022). Data for this study were collected from participants in China between February and March 2025. During this phase, although people’s daily lives and economic activities were gradually returning to normal, the consequences of the pandemic continued to affect people’s health (Mokaya et al., 2025). Mental recovery has lagged behind physical recovery (Grosso, 2025), which makes it necessary to further investigate the factors influencing mental health in the post-pandemic era.

Clinical trials have shown that the physiological effects of long COVID can persist for months to years, with a wide range of clinical manifestations, and are shaped by multiple factors including individual biology, viral-load exposure, and differences in symptom profile (De Luca et al., 2022). In particular, core symptom clusters, such as neurocognitive dysfunction and persistent fatigue syndrome, remained clinically relevant through 12 months of follow-up, and their severity was associated with the underlying comorbidity index and the intensity of the inflammatory response during the acute phase, indicating the need to establish a continuous, multidisciplinary rehabilitation support system (Hennemann et al., 2024).

Research on mental health in the post-pandemic era has revealed significant temporal heterogeneity in the prevalence of anxiety symptoms among health workers. At the start of the pandemic, a global multicenter study showed an anxiety prevalence of 44.6% among healthcare professionals. However, a systematic review conducted later in the pandemic showed a decrease to 22.8% (Khanlari et al., 2025), suggesting that strengthening social support and the normalization of the pandemic may alleviate anxiety in this population. However, variations in individual mental adjustment mechanisms still lead to a significant subgroup risk: in a cohort study of Chinese health workers, 5.2% experienced moderate to severe mental distress during the post-pandemic period. A meta-analysis further revealed a fluctuating psychological trajectory in 16.7%, with non-linear fluctuations in anxiety levels throughout repeated epidemics (Zhang et al., 2025).

Recent research has shown that economic foundation and job stability influence mental health in two ways. Several studies have shown that financial hardship and job insecurity can lead to negative feelings, such as anxiety and depression, and reduced satisfaction and wellbeing. Longitudinal studies have shown that those who were unemployed for 6 months were more likely to suffer from depression and that returning to work can provide only short-term relief from anxiety, while long-term financial insecurity has a negative effect on mental health (Crowe and Butterworth, 2016). Data from the labor market further confirm that the pace of economic recovery and mental health recovery is lagging and that there is a complex link between economic hardship and mental health (Godinić et al., 2020). Unemployment and lower income are direct causes of mental stress. Economic uncertainty, in contrast, adds to psychological problems through social identity disruption and job insecurity (Nguyen et al., 2025), and there is a significant association between economic instability and higher levels of depressive symptoms (Durizzo et al., 2022).

Perceived social support refers to the material and spiritual help and support that an individual receives from others in their social life (Zimet et al., 1990), which can alleviate an individual’s stress and enhance their coping ability, thus positively affecting mental health. On the other hand, perceived stress is an individual’s cognitive perception and evaluation of stressors (Cohen et al., 1983). There may be differences in how different individuals perceive the same stressor, and such differences can affect the impact of stress on mental health. Family support was found to reduce anxiety symptoms by 38% by decreasing stress perception, but the mediating effect of friend support was not significant (Yildirim, 2019), suggesting a specific protective mechanism for the intimacy dimension (Towner et al., 2022). However, the popularity of virtual socialization has altered traditional support patterns (Wu et al., 2025). In a longitudinal study across 12 countries, daily virtual interaction with a group resulted in a lower perception of the real world and increased stress levels (Prescott et al., 2020). Community support networks have proved resilient in the aftermath of the pandemic, as demonstrated by a community mutual assistance program in Wuhan, China, which aimed to reduce depression by increasing social capital (Wang, 2023).

Studies during the pandemic have further shown that perceived social support has a chain-mediation effect through increased psychological resilience, reducing the risk of PTSD by 24%. Perceived social support moderated the relationship between emotion regulation and health-related quality of life (QOL) in cancer patients, and the effect of emotion regulation on health-related QOL was largely determined by the perception of social support (Yang et al., 2025). These findings also suggest that perceived social support has a positive effect on health-related QOL. The findings highlight the importance of perceived social support and emotional adjustment, particularly cognitive reevaluation, in the improvement of health-related QOL in cancer patients.

Cognitive evaluation is a factor that associates stressors with experienced distress, especially distress associated with psychological and physical symptoms. Stress perception is a psychological response triggered by an individual’s cognitive appraisal of a stressor as threatening; it involves subjectively assessing the threat level of a stressful event and integrating available coping resources to meet situational requirements. In the post-pandemic era, the cognitive load has increased due to information overload and cognitive bias. On the one hand, the flood of information on social media has increased the frequency of catastrophic evaluation of health threats, and on the other hand, the persistence of negative emotions has weakened individuals’ ability to regulate perceived stress. Moreover, the cognitive fixation of negative emotions can interfere with stress regulation. These two mechanisms indicate that perceived stress is not only a critical intermediary between stressors and mental health but also a key factor in the dynamic interaction between the external environment and inner cognitive resources (Obbarius et al., 2021). The fear of missing out triggered by social media during the pandemic has added to the sense of economic pressure, and global research has shown that people who are often exposed to negative news about the outbreak experience higher levels of stress (Yang et al., 2025). However, cognitive reappraisal strategies can effectively reverse this effect: among cancer patients, high perceived social support combined with positive cognitive therapy was associated with increased health-related quality of life (Hirschmiller et al., 2025). Research has shown that high perceived stress and low perceived social support may increase the impact of occupational stressors on mental health symptoms (Zou et al., 2022; Yang et al., 2025).

In the social buffering hypothesis (Cohen and Wills, 1985), perceived social support has been found to act as a buffer in stressful situations and can reduce the negative impact of stress on an individual’s mental health. Individuals with higher perceived social support are more likely to cope with stress and report lower subjective assessments of stressful events, thus reducing the negative impact of stress. Perceived social support can improve an individual’s ability to cope with stress and reduce the perceived severity of stress by providing emotional support and practical help (Qin et al., 2022).

Psychological resilience, a core element of psychological adaptation, refers to the ability to cope with challenges and recover from adverse circumstances (Masten, 2001). The quality-stress model (Fraley and Roisman, 2019) suggested that psychological resilience protects adolescents from the deleterious effects of adverse developmental events or stressors on healthy psychological development. Research has shown that mental resources such as resilience can significantly reduce fear and mental distress (Li et al., 2025). Research has shown that psychological resilience plays an important role in mitigating the adverse effects of stress on the mental health of healthcare workers (Yildirim, 2019). Perceived risk and challenge stressors have different moderating effects on the impact of psychological resilience (Xie et al., 2023).

Based on this, the following theoretical hypotheses are proposed in this study:

1. Perceived social support is negatively associated with general psychological distress.
2. Perceived social support is negatively associated with perceived stress, and perceived stress mediates the association between perceived social support and general psychological distress.
3. Psychological resilience moderates the direct association between perceived social support and general psychological distress. Specifically, the negative association between perceived social support and general psychological distress will be stronger among individuals with higher psychological resilience.
4. Psychological resilience moderates the association between perceived social support and perceived stress. Specifically, the negative association between perceived social support and perceived stress will be stronger among individuals with higher psychological resilience.

Based on the stress-buffering hypothesis (Cohen and Wills, 1985), this study tests a moderated mediation model in which psychological resilience concurrently moderates both the direct association between perceived social support and general psychological distress and the predictive path from perceived social support to perceived stress. This model helps examine the pathway linking perceived social support to general psychological distress and highlights the protective role of personal psychological resources in stress coping. The results support differentiated interventions: strengthening perceived social support to compensate for inadequate coping resources among low-resilience individuals and delivering cognitive reappraisal-focused cognitive-behavioral interventions to reduce general psychological distress among individuals with high perceived stress.

## 2 Objects and methods

### 2.1 Objects of the study

This study adopted a cross-sectional survey design with convenience sampling. Data were collected between February and March 2025 via Questionnaire Star, with questionnaires disseminated through WeChat. Questionnaires were distributed to the general population across mainland China. Eligible participants were individuals born and residing in China who were able to read Chinese. A total of 12,241 questionnaires were collected; after excluding responses with logical inconsistencies (i.e., participants with mismatched answers to two duplicate items), 8,131 valid questionnaires were retained for analysis. Written informed consent was obtained from both the minor participants and their legal guardians, while electronic informed consent was obtained from adult participants prior to questionnaire completion, and those who completed the survey were entered into a lottery to receive WeChat red envelopes (RMB 1–3) as an incentive. This study was reviewed and approved by the Medical Ethics Committee of the First Affiliated Hospital of Air Force Medical University (Approval No.: KY20234187-1, 2023).

### 2.2 Research tools

A basic information questionnaire was used to collect demographic information about the participants, including gender, age, education, marital status, occupation, annual per capita household income, place of residence, and whether their working lives were directly affected by the pandemic.

Perceived social support was measured using the Chinese version of the Multidimensional Scale of Perceived Social Support (MSPSS), which has demonstrated good reliability in Chinese populations (Liang et al., 2019). The scale consists of 12 items across three dimensions: family support, friend support, and other support. The scale is rated on a 7-point scale from 1 to 7, and the total score ranges from 12 to 84, with higher scores indicating higher levels of perceived social support (Adesola et al., 2025). Cronbach’s alpha coefficient of the scale in this study was 0.98.

The Chinese Perceived Stress Scale (CPSS), with good reliability in Chinese populations (She et al., 2021), was used to assess the participants’ perceived stress. The scale consists of 14 items, comprising two dimensions: tension and loss of control. It is scored on a 5-point scale from 1 to 5, with the loss of control dimension being reverse-scored, and the total score ranges from 14 to 60, with higher total scores indicating a higher level of perceived stress (Shi et al., 2019). In this study, the scale’s Cronbach’s alpha coefficient was 0.933.

The Chinese version of the Connor–Davidson Resilience Scale (CD-RISC-10), with good psychometric properties in Chinese populations (Liang et al., 2020), was used to assess the participants’ psychological resilience. The scale consists of 10 items. A higher total score indicates a higher level of psychological resilience (Ye et al., 2017). In this study, Cronbach’s alpha coefficient for this scale was 0.987.

General psychological distress was screened using the Chinese version of the 28-item General Health Questionnaire (GHQ-28), which has demonstrated good reliability in Chinese populations (Liu et al., 2020). The scale contains four dimensions, namely somatic symptoms, anxiety/insomnia, social dysfunction, and severe depression, with seven questions for each dimension. A 4-point scale from 1 to 4 was used. Of these, the anxiety/insomnia and social dysfunction dimensions were reverse-scored. The total score ranges from 28 to 112, with a higher total score indicating a higher level of general psychological distress and poorer mental health compared with that in the previous weeks (Sterling, 2011). In this study, Cronbach’s alpha coefficient for this scale was 0.932.

## 3 Statistical methods

Descriptive statistics were computed for demographic information and study variables. Normally distributed quantitative data were presented as mean ± standard deviation (M ± SD). Independent-samples *t*-tests were used to examine differences in perceived social support, psychological resilience, perceived stress, and general psychological distress according to gender, income, marital status, and occupation. Pearson’s product–moment correlation coefficients were used to assess bivariate correlations among the study variables.

The moderated mediation model was established using PROCESS macro 4.3, Model 8. Bias-corrected bootstrapping with 5,000 resamples was used; a 95% CI excluding zero indicated a statistically significant moderated mediation effect. In Model 8, perceived social support served as the independent variable, perceived stress as the mediator, and general psychological distress as the dependent variable. Psychological resilience was the moderator, exerting moderating effects on two paths: the path from perceived social support to perceived stress and the path from perceived social support to general psychological distress. Covariates including gender, income, marital status, and occupation were controlled in Model 8, and the interaction term of perceived social support × psychological resilience was included. All continuous study variables were standardized (Z-scored) (Hayes and Rockwood, 2017).

To examine demographic heterogeneity in the association between perceived stress and general psychological distress, exploratory simple moderation analyses were conducted as a supplement to the primary hypothesized model. Gender, income, marital status, and occupation were selected as moderators. Hayes’ PROCESS Model 1 was adopted to estimate moderating effects, with perceived social support, psychological resilience, and the remaining demographic covariates controlled. Given the large sample size of this study, high statistical power may detect statistically significant yet practically weak interaction effects. For this reason, only interaction terms are reported, and simple slope decomposition and subgroup effect comparisons are not performed.

Confirmatory factor analysis (CFA) for assessing convergent and discriminant validity, as well as variance inflation factor (VIF) and tolerance calculations for multicollinearity diagnosis, was performed using RStudio-2026.08.2–200. The lavaan package was used for CFA to test the two-factor and competing one-factor measurement models. Variance inflation factor and tolerance statistics were computed using a linear regression analysis to evaluate multicollinearity among key predictors. SPSS 21.0 with PROCESS macro 4.3 was used for descriptive statistics, *t*-tests, correlation analysis, moderated mediation and simple moderation analyses, bootstrap tests, and Harman’s single-factor test. The significance level was set at α = 0.05 (Podsakoff et al., 2010).

## 4 Results

### 4.1 Common method bias test

This study assessed common method bias using Harman’s one-factor test and extracted potential factors using principal component analysis (PCA). The results of exploratory factor analysis (EFA) showed that six principal components were identified in the data. The explained variance of the first factor was 45.32%. Although this value is below the commonly used 50% threshold (Shneif, 2015), Harman’s single-factor test is a diagnostic screening procedure rather than a definitive test; accordingly, common-method bias cannot be entirely ruled out given the same-source, self-report, cross-sectional design.

### 4.2 Demographic profile

In terms of demographic characteristics, the distribution of the sample is as follows: the sample was predominantly male (74%), followed by female (26%) participants; the 18–20-year-old age group accounted for the largest proportion (53.1%), with students (28.4%) and employees of public institutions (26.3%) constituting the main occupational groups. The majority of participants were single (77.1%), and low-income families accounted for the largest proportion of the sample (54.5%).

### 4.3 Group differences in study variables by demographic variables

Group differences across demographic subgroups are presented in Supplementary Table S1. Independent-samples *t*-tests revealed significant between-group mean differences in the majority of study variables across gender, income, marital status, and occupation subgroups (all *p* < 0.05). The corresponding *t* statistics and Cohen’s *d* effect sizes are summarized in the table. In terms of effect magnitudes, gender yielded moderate-to-large Cohen’s *d* values for general psychological distress, psychological resilience, and perceived social support. Marital status and occupation mostly produced small to nearly moderate effects, while income generally yielded trivial effect sizes, suggesting statistically significant but practically limited mean differences.

### 4.4 Bivariate correlation between the study variables

General psychological distress was positively correlated with perceived stress (*r* = 0.59, *p* < 0.01) and negatively correlated with psychological resilience and perceived social support (*r* = −0.59, *p* < 0.01 and *r* = −0.60, *p* < 0.01). In addition, there was a significant positive correlation between psychological resilience and perceived social support (*r* = 0.891, *p* < 0.01). The relevant results are shown in Table 1.

**Table 1:** Correlations between general psychological distress, perceived stress, psychological resilience, and perceived social support.

Variables	M ± SD	GHQ-28	CPSS	CD-RISC-10	MSPSS
General psychological distress, GHQ-28	62.03 ± 10.34	–	–	–	
Perceived stress, CPSS	38.42 ± 7.03	0.59**	–	–	
Psychological resilience, CD-RISC-10	27.82 ± 13.01	−0.59**	−0.68**	–	
Perceived social support, MSPSS	47.08 ± 22.81	−0.60**	−0.61**	0.891**	–

**p < 0.01.

### 4.5 Measurement model and multicollinearity check

Confirmatory factor analysis (CFA) was performed to evaluate the discriminant validity between perceived social support and psychological resilience. The two-factor measurement model yielded acceptable fit indices: *χ*^2^(208) = 10240.65, CFI = 0.973, TLI = 0.970, RMSEA = 0.077, and SRMR = 0.012. Convergent validity was supported, with AVE > 0.50 and CR > 0.70 for both constructs. The square roots of AVE for perceived social support and psychological resilience exceeded their latent correlation (*r* = 0.891), satisfying the Fornell–Larcker criterion. The competing one-factor model demonstrated poor fit and was significantly worse than the two-factor model, demonstrating an empirical distinction between the two constructs.

Variance inflation factor (VIF) and tolerance values were calculated to examine multicollinearity in the regression model. The VIF values of perceived social support and psychological resilience were 5.065 and 5.784, respectively, with tolerance values of 0.197 and 0.173. All predictors had VIF < 10 and tolerance >0.10, indicating no serious multicollinearity.

Therefore, perceived social support and psychological resilience were considered distinct constructs.

### 4.6 Moderated mediation model testing

Perceived stress significantly and positively predicted general psychological distress (*β* = 0.3628, *p* < 0.001), which satisfied the basic condition for the mediation relationship (Figure 1).

**Figure 1:**
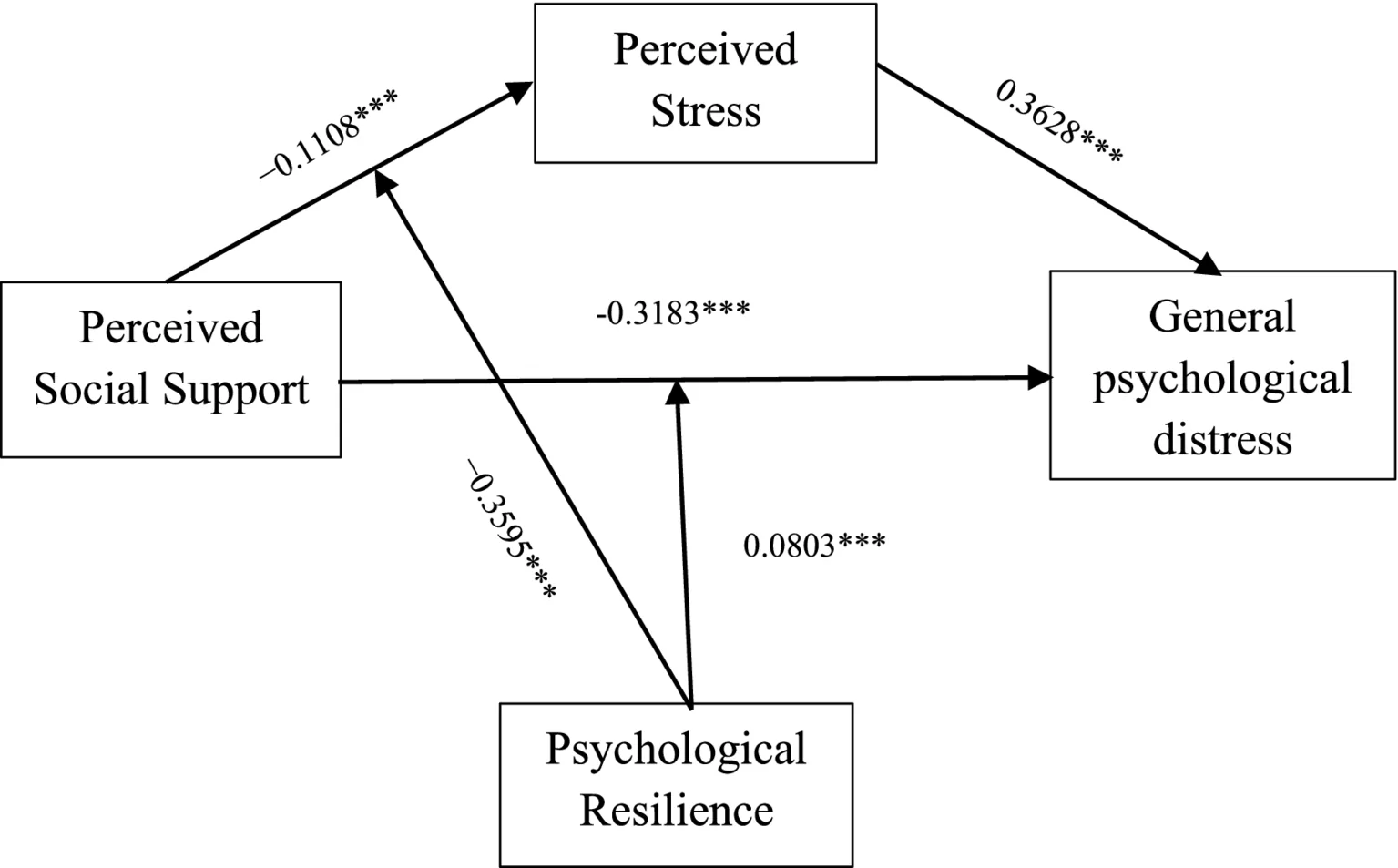
Standardized path diagram of Hayes’ PROCESS Model 8 (first-stage dual moderated mediation model). Values represent standardized beta coefficients. Paths from psychological resilience denote X × W interaction terms. ***p < 0.001. Covariates (gender, income, occupation, and marital status) were controlled. All continuous variables were z-standardized.

In the regression model for perceived stress ( $R^{2}$ = 0.5431, $F_{7,8123}$ = 1379.4709, *p* < 0.001), the interaction between perceived social support and psychological resilience was statistically significant ( β = − 0.3595, *p* < 0.001, Δ*R*^2^ = 0.0776, $F_{1,8123}$ = 1380.4136, *p* < 0.001), indicating that psychological resilience significantly moderated the path from perceived social support to perceived stress (Figure 1). Simple slope analysis revealed a reversed pattern across resilience levels. At low psychological resilience (*M* − 1*SD*, β = 0.2488, *p* < 0.001), perceived social support positively predicted perceived stress. In contrast, the association became negative and significant at high resilience (*M* + 1*SD*, β = − 0.4703, *p* < 0.001) (Figure 2).

**Figure 2:**
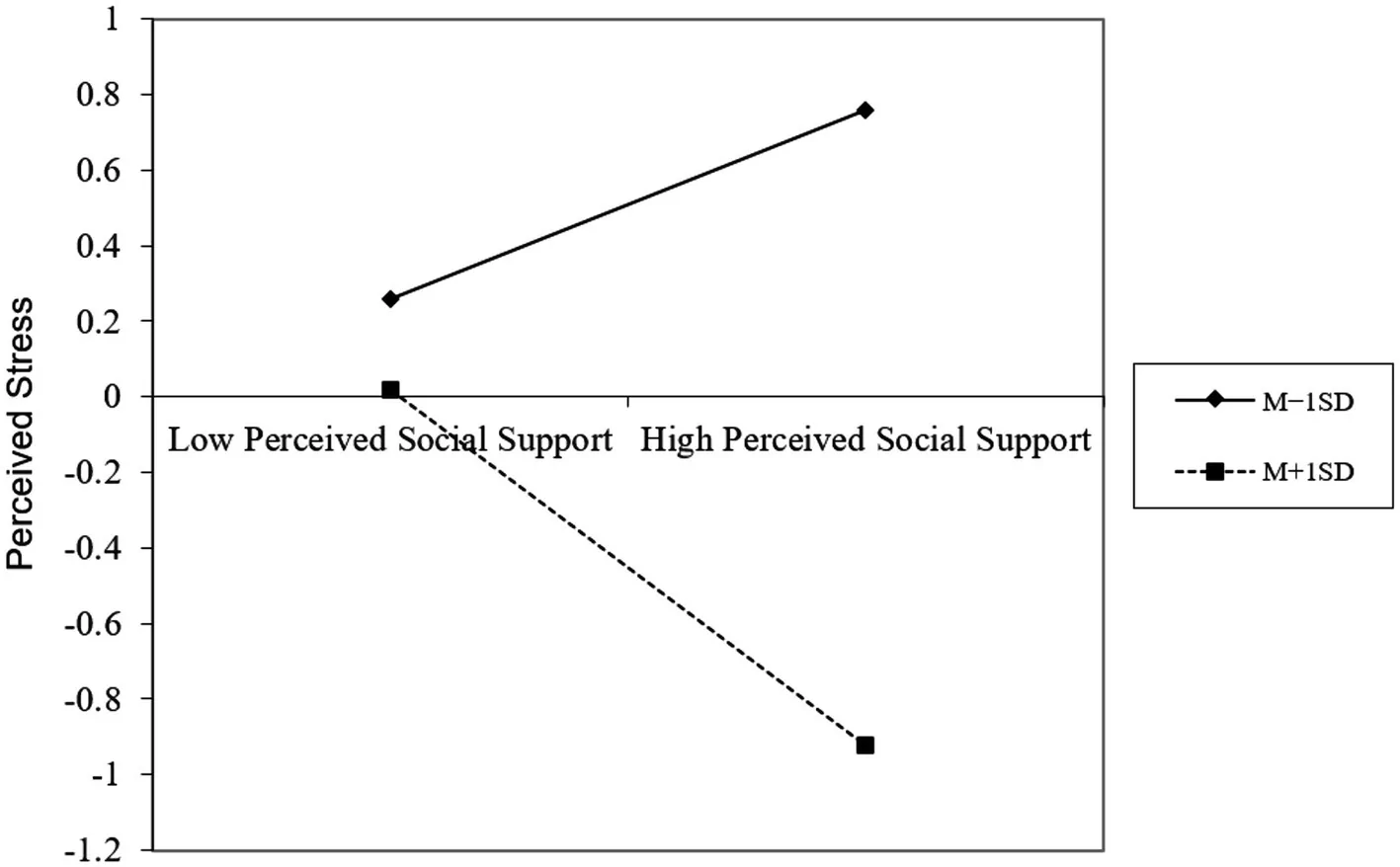
Moderation of the association between perceived social support and perceived stress.

In the model predicting general psychological distress ( $R^{2}$ = 0.4859, $F_{8,8122}$ = 959.7436, *p* < 0.001), the interaction term was significant ( β = 0.0803, *p* < 0.001, Δ*R*^2^ = 0.0033, $F_{1,8122}$ = 52.3231, *p* < 0.001), confirming that psychological resilience moderated the direct effect (Figure 1). Simple slope analysis showed that the protective direct effect of social support was significant at both low and high resilience levels but gradually attenuated as resilience increased: low resilience ( M−1SD,β = − 0.3987, *p* < 0.001) and high resilience ( M+1SD,β = − 0.2380, *p* < 0.001) (Figure 3).

**Figure 3:**
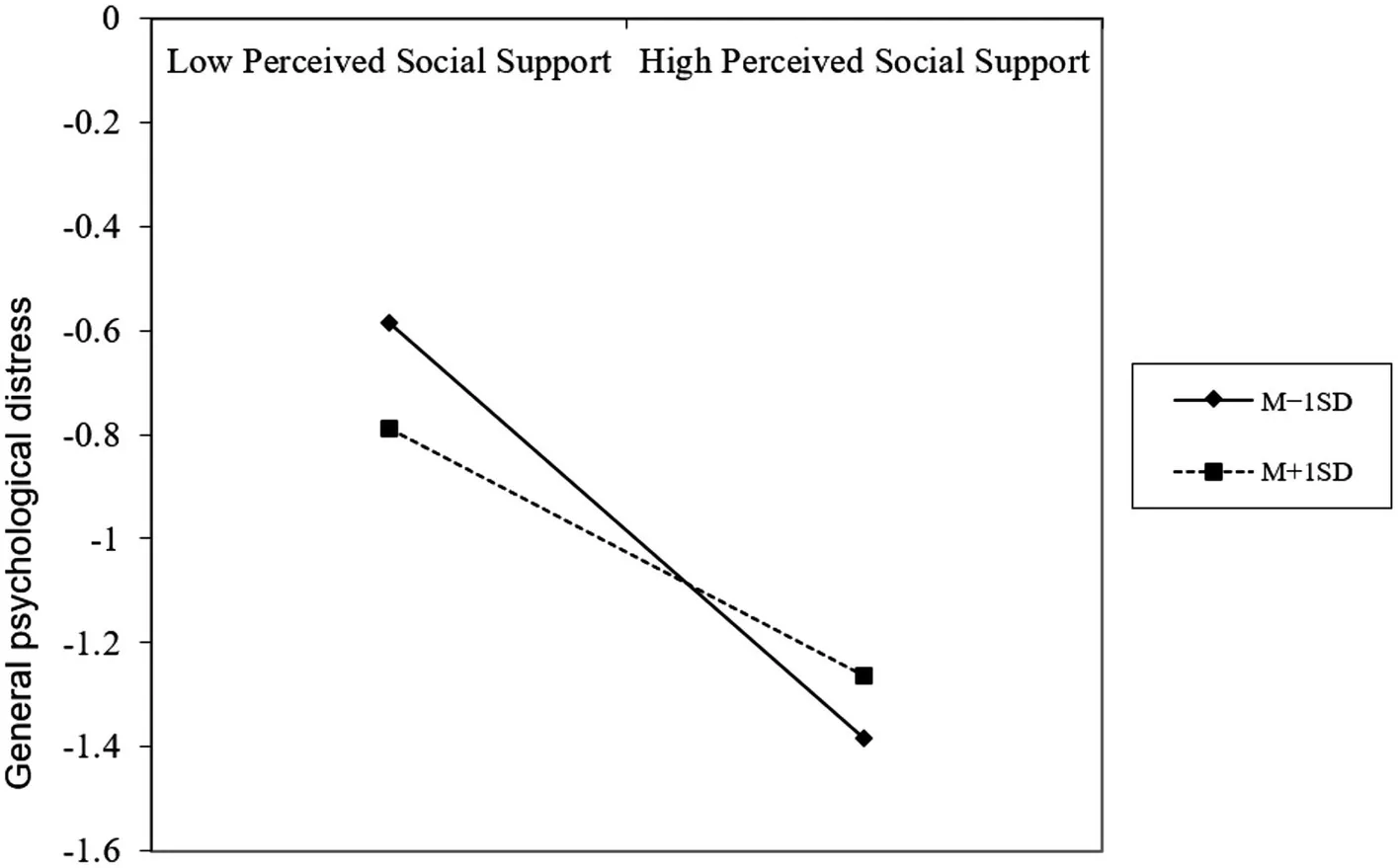
Moderation of the direct effect of perceived social support on general psychological distress.

The conditional indirect effect of perceived social support on general psychological distress through perceived stress was statistically significant at both low and high levels of psychological resilience. Specifically, the indirect effect was positive and significant under low resilience [*M* − 1*SD*, β = 0.0903, bootstrap 95%CI (0.0716, 0.1105)], whereas a significant negative indirect effect was observed under high resilience [ M+1SD , β = − 0.1706, bootstrap 95%CI (−0.1970, −0.1439)]. The index of moderated mediation was significant [index = −0.1304, bootstrap 95%CI (−0.1449, −0.1165)], demonstrating that the magnitude and direction of the indirect effect significantly varied as a function of psychological resilience. Taken together, the hypothesized first-stage moderated mediation model was fully supported.

### 4.7 Exploratory simple moderation analyses

The interaction between perceived stress and income was significant (*β* = 0.055, *p* < 0.001, Δ*R*^2^ = 0.0007), as was the interaction between perceived stress and marital status (*β* = 0.168, *p* < 0.001, Δ*R*^2^ = 0.0024), and the interaction between perceived stress and gender was significant (*β* = 0.085, *p* < 0.001, Δ*R*^2^ = 0.0007). The interaction between perceived stress and occupation was non-significant (*β* = 0.017, *p* = 0.303, Δ*R*^2^ = 0.0001). Accordingly, income, marital status, and gender weakly moderated the association between perceived stress and general psychological distress, whereas no moderating effect was found for occupation. Considering the large sample size, the significant interactions exhibited extremely small incremental explained variance, indicating statistically significant but practically limited effects.

## 5 Discussion

This study examined the associations between perceived social support, perceived stress, psychological resilience, and general psychological distress in a large Chinese sample surveyed in the post-pandemic period. Several patterns emerged. Consistent with the social support buffering hypothesis, perceived social support was associated with lower general psychological distress, and perceived stress statistically accounted for part of this association. Perceived social support may broaden individuals’ coping repertoires and reframe negative cognitive appraisals, which is compatible with evidence that perceived social support is indirectly associated with wellbeing through lower stress perception (Kim et al., 2025; Luo et al., 2023). Online social networks may partly compensate for the psychological consequences of physical isolation by maintaining emotional connections (Jahangir et al., 2025). These patterns support the potential value of strengthening social support networks as one component of population mental health promotion, particularly among individuals with circumscribed support networks, such as those on low incomes or in precarious occupations.

Perceived stress is the subjective appraisal of stressors as threatening, and it was consistently the strongest correlate of general psychological distress in this sample (*r* = 0.59). Individuals who appraised events as more threatening tended to report higher general psychological distress, which aligns with prior evidence linking elevated perceived stress to depressive symptomatology (Kirchner et al., 2025). Cognitive-behavioral approaches that help individuals reappraise stressors may therefore be a reasonable target for future intervention trials in individuals with high perceived stress.

A key finding was the significant crossover interaction between perceived social support and psychological resilience in predicting perceived stress (*β* = −0.3595, *p* < 0.001). At high levels of psychological resilience, greater perceived social support was associated with lower perceived stress, as expected; at low levels of psychological resilience, however, the conditional indirect effect reversed direction and became positive. This is consistent with a previous study (Yılmaz and Çıtak, 2025), which likewise observed that the benefit of social support was not uniform across individuals. Additionally, research has shown that the downstream effects of support depend on personal resources (Zhao et al., 2023), and a multilevel study in early adolescents similarly found non-linear combinations of psychological resilience and perceived social support in relation to mental health (Zhu et al., 2024). One interpretation is that individuals with low psychological resilience may lack the cognitive or behavioral resources to convert received support into stress reduction and may even experience social demands associated with support as an additional burden. This possibility should be examined with longitudinal designs before it is treated as a firm mechanism.

Psychological resilience also significantly moderated the direct association between social support and general psychological distress (*β* = 0.0803, *p* < 0.001), although the incremental variance explained was small (Δ*R*^2^ = 0.0033), so the practical magnitude of this moderation should not be overstated. Taken together with the first-stage moderation, the pattern is broadly compatible with resource-based models of resilience, which propose that personal resources shape how external support is translated into wellbeing (Weedage et al., 2025). We caution, however, that these are associations estimated from cross-sectional self-report data and do not establish that psychological resilience causally converts perceived social support into perceived stress regulation.

Exploratory subgroup analyses indicated that the stress-distress association was further moderated by gender, income, and marital status but not by occupational stability. Women, lower-income individuals, and married individuals appeared more vulnerable to distress associated with high perceived stress. Gender differences may reflect socialization, coping repertoires, and role demands (Graves et al., 2021), while income-related differences are consistent with the broader literature linking economic strain to worse mental health. These exploratory findings were statistically significant but small in effect size (Δ*R*^2^ <= 0.0024) and should be treated as hypothesis-generating rather than as precise subgroup estimates.

Several limitations qualify these findings. The sample was predominantly male (74.0%) and young (53.1% aged 18–20 years, and with a large proportion of students); it should therefore not be regarded as representative of the Chinese general population, and digital-access and self-selection biases cannot be ruled out. Next, all variables were self-reported by the same respondents at the same time, raising the possibility of common-method variance. In addition, a CFA comparing perceived social support and psychological resilience showed that some residual overlap between these two constructs should nevertheless be kept in mind. Additionally, the data were collected in a single national convenience sample at one point in time; temporal ordering among the study variables cannot be inferred, and no causal mechanism should be claimed.

In the future, longitudinal designs are needed to confirm the direction of the observed crossover interaction and to test whether the moderating role of psychological resilience generalizes across occupational groups and different stress contexts, including post-pandemic student populations (Foster et al., 2025).

## 6 Conclusion

In summary, in this cross-sectional survey conducted between February and March 2025, perceived social support was associated with lower general psychological distress, partly through lower perceived stress. Psychological resilience significantly moderated both the path from perceived social support to perceived stress and the path from perceived social support to general psychological distress. Notably, the conditional indirect effect reversed sign across resilience levels, becoming positive among individuals with low psychological resilience.

## Data Availability

The datasets generated during this study are available from the corresponding author upon reasonable request. Requests to access the datasets should be directed to Shengjun Wu, wushj@fmmu.edu.cn.
