# Supplementary material for: Perceived social support and general psychological distress in the post-pandemic era: mediation of perceived stress and moderation of psychological resilience
Source: Front Psychol. 2026 Oct 12;17:1939954. doi: 10.3389/fpsyg.2026.1939954 (PMC13701669; doi:10.3389/fpsyg.2026.1939954)
Supplement: Supplementary file 1 [file Table_1.pdf]

Table S1 Group differences in study variables by demographic variables

Variables	Demographic Variables		*M* ± *SD*	*t/F*	*p*	Cohen's *d*
General psychological distress	gender	male (*N* = 6020)	60.22 ± 10.34	-27.87	0.000***	0.70
		female (*N* = 2111)	67.19 ± 8.46			
	income	low (*N* = 4430)	60.82 ± 10.34	-11.61	0.000***	0.26
		high (*N* = 3701)	63.47 ± 10.16			
	marital status	single (*N* =6270)	60.39 ± 10.29	-27.37	0.000***	0.72
		married (*N* =1861)	67.54 ± 8.43			
	occupation	stable (*N* = 2816)	63.19 ± 10.03	-14.08	0.000***	0.33
		unstable (*N* = 5315)	69.44 ± 6.51			
Perceived Stress	gender	male (*N* = 6020)	37.51 ± 7.49	-20.20	0.000***	0.51
		female (*N* = 2111)	41.01 ± 4.59			
	income	low (*N* = 4430)	38.03 ± 7.25	-5.42	0.000***	0.12
		high (*N* = 3701)	38.88 ± 6.73			
	marital status	single (*N* =6270)	37.67 ± 7.45	-17.97	0.000***	0.47
		married (*N* =1861)	40.94 ± 4.53			
	occupation	stable (*N* = 2816)	37.14 ± 7.69	-12.08	0.000***	0.28
		unstable (*N* =5315)	39.10 ± 6.55			
Psychological Resilience	gender	male (*N* = 6020)	30.35 ± 12.96	31.34	0.000***	0.79
		female (*N* = 2111)	20.61 ± 10.16			
	income	low (*N* = 4430)	28.33 ± 13.09	3.84	0.000***	0.09
		high (*N* = 3701)	27.22 ± 12.89			
	marital status	single (*N* =6270)	29.11 ± 13.19	16.68	0.000***	0.44
		married (*N* =1861)	23.48 ± 11.37			
	occupation	stable (*N* = 2816)	31.51 ± 13.16	19.02	0.000***	0.44
		unstable (*N* = 5315)	25.87 ± 12.50			
Perceived Social Support	gender	male (*N* = 6020)	51.72 ± 22.34	32.97	0.000***	0.83
		female (*N* = 2111)	33.85 ± 18.55			
	income	low (*N* = 4430)	47.65 ± 22.89	2.50	0.013*	0.06
		high (*N* = 3701)	46.39 ± 22.70			
	marital status	single (*N* =6270)	49.08 ± 23.04	14.71	0.000***	0.39
		married (*N* =1861)	40.34 ± 20.64			
	occupation	stable (*N* = 2816)	53.49 ± 22.59	18.84	0.000***	0.44
		unstable (*N* = 5315)	43.68 ± 22.19			

Note: **p* < 0.05, ****p* < 0.001.
